# An expression signature of syndecan-1 (CD138), E-cadherin and c-met is associated with factors of angiogenesis and lymphangiogenesis in ductal breast carcinoma *in situ*

**DOI:** 10.1186/bcr1641

**Published:** 2007-01-23

**Authors:** Martin Götte, Christian Kersting, Isabel Radke, Ludwig Kiesel, Pia Wülfing

**Affiliations:** 1 Department of Obstetrics and Gynecology, Münster University Hospital, Domagkstrasse 11, Münster, D-48149, Germany; 2 Department of Pathology, Münster University Hospital, Domagkstrasse, Münster, D-48149, Germany

## Abstract

**Introduction:**

Heparan sulphate proteoglycan syndecan-1 modulates cell proliferation, adhesion, migration and angiogenesis. It is a coreceptor for the hepatocyte growth factor receptor c-met, and its coexpression with E-cadherin is synchronously regulated during epithelial-mesenchymal transition. In breast cancer, changes in the expression of syndecan-1, E-cadherin and c-met correlate with poor prognosis. In this study we evaluated whether coexpression of these functionally linked prognostic markers constitutes an expression signature in ductal carcinoma *in situ *(DCIS) of the breast that may promote cell proliferation and (lymph)angiogenesis.

**Methods:**

Expression of syndecan-1, E-cadherin and c-met was detected immunohistochemically using a tissue microarray in tumour specimens from 200 DCIS patients. Results were correlated with the expression patterns of angiogenic and lymphangiogenic markers. Coexpression of the three prognostic markers was evaluated in human breast cancer cells by confocal immunofluorescence microscopy and RT-PCR.

**Results:**

Coexpression and membrane colocalization of the three markers was confirmed in MCF-7 cells. E-cadherin expression decreased, and c-met expression increased progressively in more aggressive cell lines. Tissue microarray analysis revealed strong positive staining of tumour cells for syndecan-1 in 72%, E-cadherin in 67.8% and c-met in 48.6% of DCIS. E-cadherin expression was significantly associated with c-met and syndecan-1. Expression of c-met and syndecan-1 was significantly more frequent in the subgroup of patients with pure DCIS than in those with DCIS and a coexisting invasive carcinoma. Levels of c-met and syndecan-1 expression were associated with HER2 expression. Expression of c-met significantly correlated with expression of endothelin A and B receptors, vascular endothelial growth factor (VEGF)-A and fibroblast growth factor receptor-1, whereas E-cadherin expression correlated significantly with endothelin A receptor, VEGF-A and VEGF-C staining.

**Conclusion:**

Syndecan-1, E-cadherin and c-met constitute a marker signature associated with angiogenic and lymphangiogenic factors in DCIS. This coexpression may reflect a state of parallel activation of different signal transduction pathways, promoting tumour cell proliferation and angiogenesis. Our findings have implications for future therapeutic approaches in terms of a multiple target approach, which may be useful early in breast cancer progression.

## Introduction

Syndecan-1/CD138 (Sdc1) is a cell surface heparan sulphate proteoglycan that is highly expressed by epithelial and plasma cells. Via its heparan sulphate chains, Sdc1 binds to a variety of growth and angiogenic factors and acts as a classical coreceptor for growth factor receptors, thus promoting cell proliferation [1]. Moreover, Sdc1 interacts with ligands in the extracellular matrix and on cell surfaces, functioning as a cell adhesion molecule [1]. We recently showed that Sdc1 is a modulator of proteolytic activities and chemokine functions *in vivo*, which orchestrates leucocyte recruitment and tissue remodelling during inflammation and wound repair [2-4]. In Sdc1-deficient and Sdc1-overexpressing mouse models, abnormal blood vessel formation is observed during wound repair, confirming a role for Sdc1 as a regulator of angiogenesis *in vivo *[2,4]. Because the biological functions of Sdc1 potentially affect several steps in tumour progression, it is not surprising that a prognostic value has been assigned to changes in Sdc1 expression in several cancer types, including colorectal, gastric, pancreatic, prostate, lung, endometrial and ovarian cancers, as well as squamous cell carcinoma of the head and neck (for review, see Yip and coworkers [5]). In breast cancer, increased expression of Sdc1 correlates with an unfavourable prognosis [6-8] and poor response to chemotherapy [9]. Of note, several proteins that are functionally linked to Sdc1 by virtue of their biology are prognostic markers on their own (Table 1). In multiple myeloma, Sdc1 mediates ligand binding and signalling through the hepatocyte growth factor (HGF) receptor tyrosine kinase c-met, resulting in increased cancer cell proliferation [10].

Similar mechanisms may be of relevance in breast cancer, because prognostic value has been established for c-met expression in a number of clinical studies of breast cancer patients (Table 1). Signal transduction mediated by c-met modulates cell dissociation and motility, and protease overexpression [11,12]. Moreover, ribozyme targeting of c-met in mammary cancer cells reduced mammary cancer and tumour-associated angiogenesis in a xenograft model [12]. To mobilize its full transforming potential in breast cancer, c-met appears to depend on coactivating factors, such as overexpression of additional proto-oncogenes (MYC, RON), or β_4 _integrin activity [13-15]. Similarly, Sdc1 regulates α_v_β_3 _integrin activation and signalling in breast cancer cell lines [16,17]. Sdc1-integrin complexes may thus synergistically contribute to tumour progression driven by c-met overexpression.

The calcium-dependent cell-cell adhesion molecule E-cadherin (E-cad) is an established prognostic marker in breast cancer (Table 1). E-cad expression is irreversibly lost in invasive lobular breast cancer, and this feature has been used by pathologists to distinguish between ductal and lobular neoplasia [18-20]. Like Sdc1, E-cad is mostly present in epithelial cells, and is required for maintaining the epitheloid phenotype and inhibition of density-dependent cell growth [21]. Coordinated regulation of E-cad and Sdc1 expression is seen during development [22] and in mammary tumour cells subjected to antisense RNA mediated downregulation of Sdc1 [23] or E-cad [24], respectively. Sdc1 and E-cad colocalize and coimmunoprecipitate with the transcriptional regulator β-catenin in epithelial cells, suggesting both a functional and physical association [25]. Of note, expression of Sdc1 is required for proper development of a β-catenin responsive mammary epithelial progenitor cell population, which appears to be the mechanistic principle underlying resistance of Sdc1-deficient mice to wnt-1 mediated breast cancer [5,26-28].

In the present study we evaluated coexpression of the three functionally linked prognostic factors, namely Sdc1, E-cad and c-met, in ductal carcinoma *in situ *(DCIS) of the breast by semiquantitative immunohistochemistry of a tissue microarray containing tumour specimens from 200 patients. We recently showed that several proangiogenic factors, including fibroblast growth factor receptor (FGFR)-1, vascular endothelial growth factor (VEGF)-C, its receptor Flt-4, and the endothelin A receptor (ET_A_R), are highly expressed in DCIS, suggesting that *in situ *carcinomas can induce angiogenesis and lymphangiogenesis [29]. Although expression of endothelin and VEGF receptors was previously believed to be largely restricted to the vascular endothelium, these receptors have also been detected in breast cancer cells, suggesting an autocrine effect of their ligands on tumour cells [29-31]. Because Sdc1 and c-met modulate angiogenesis *in vivo *[2,4,12,32,33], we determined the association of these molecules with markers of angiogenesis and lymphangiogenesis in DCIS. Moreover, we characterized the coexpression of Sdc1, E-cad and c-met in three differently aggressive human breast cancer cell lines by RT-PCR and by confocal immunofluorescence microscopy. The aim of the study was to determine whether coexpression of the functionally linked molecular markers Sdc1, E-cad and c-met may constitute a novel angiogenesis-associated molecular marker signature in DCIS.

## Materials and methods

### Cell culture and confocal immunofluorescence microscopy

The human breast cancer cell lines MCF-7 and MDA-MB468 were from our cell culture collection [34]. MCF-7 cells were cultured in RPMI-1640 medium (Gibco, Karlsruhe, Germany) supplemented with 10% foetal calf serum (PAA, Cölbe, Germany) and 2 mmol/l L-glutamine. MDA-MB231 (CLS Cell Lines Service, Eppelheim, Germany) and MDA-MB468 cells were maintained in DMEM (Dulbecco's modified Eagle medium) high-glucose medium (Invitrogen, Karlsruhe, Germany) supplemented with 1% L-glutamine, 1% penicillin-streptomycin and 10% foetal calf serum at 7.5% carbon dioxide. For immunocytochemistry, cells were grown to 80% confluence in eight-well chamber slides (Corning Costar, Wiesbaden, Germany), fixed with ice-cold methanol, and subjected to immunostaining as follows. After a 30 min blocking step with phosphate-buffered saline (PBS) containing 10% goat serum (DAKO, Glostrup Denmark), the slides were washed with PBS, and the following primary antibodies were used in an overnight incubation at 4°C at a dilution of 1:100 in PBS/1% bovine serum albumin: mouse anti-syndecan-1 (clone BB4; Serotec, Duesseldorf, Germany), rabbit anti-c-met (Santa Cruz, Santa Cruz, CA, USA), and mouse anti-E-cadherin (BD Transduction Laboratories, San Diego, CA, USA). The slides were washed three times for 5 min with PBS and incubated with AlexaFluor 546-conjugated goat-anti-mouse IgG (Invitrogen) and AlexaFluor 488 conjugated donkey-anti-rabbit IgG (Invitrogen) secondary antibodies in PBS/1% bovine serum albumin at a dilution of 1:600 at room temperature for 30 min. Samples that were incubated without a primary antibody served as controls. Slides were washed three times for 5 min with PBS and mounted using VectaShield (Vector Labs, Burlingame, CA, USA). Laser scanning microscopy was performed using a Leica TCS SL confocal microscope (Leica, Bensheim, Germany).

### RT-PCR analysis

For RT-PCR analysis, total cellular RNA was prepared from MCF-7, MDA-MB468, and MDA-MB231 cells using the RNeasy Mini kit (Qiagen, Hilden, Germany) in the presence of RNAse-free DNAse. Total RNA 1 μg was reverse transcribed using the Advantage RT-for-PCR Kit (BD Clontech, Heidelberg, Germany) and amplified in a BioMetra PCR reactor (BioMetra, Göttingen, Germany). A DNA fragment corresponding to base pairs 1233–1303 of human Sdc-1 mRNA (accession # NM_001006946.1) was amplified using the primers 5'-AGGACGAAGGCAGCTACTCCT-3' and 5'-TTTGGTGGGCTTCTGGTAGG-3' and the following amplification cycle: 94°C for 4 min; 27 cycles of 94°C for 30 s, 60°C for 30 s and 72°C for 30 s; and 72°C for 5 min. The primers 5'-TGGGTTATTCCTCCCATCAG-3' and 5'-TTTGTCAGGGAGCTCAGGAT-3' were used to amplify a PCR fragment corresponding to base pairs 590–1066 of human E-cad (accession # NM_004360.2) and the following amplification cycle: 94°C for 5 min; 25 cycles of 94°C for 60 s, 60°C for 60 s, 72°C for 60 s; and 72°C for 10 min. Using the same amplification protocol, the primers 5'-TTCGGGTTGTAGGAGTCTTCT-3' and 5'-GGTTGCTGATTTTGGTCTTGC-3' were used to amplify base pairs 3844–4105 of the human c-met mRNA (accession # NM_000245.2). The housekeeping gene β-actin was used as an internal control and amplified using the primers 5'-CAAAGACCTGTACGCCAACAC-3' and 5'-CATACTCCTGCTTGCTGATCC-3', corresponding to base pairs 943–1220 of human β-actin (accession # NM_001101) and the following amplification cycle: 95°C for 3 min; 18 cycles of 95°C for 1 min, 56°C for 1 min and 72°C for 1 min; and 72°C for 10 min.

PCR products were subjected to gel electrophoresis on 1.5% agarose gels. After ethidium bromide staining, DNA bands were photographed under UV illumination using a BioDoc Analyze system (Biometra). Scanned bands were analyzed using the Image J software (NIH, Bethesda, MA, USA) and signal intensities were normalized for actin expression.

### Patients and tumour samples

To study expression and interrelation of c-met, Sdc1, and E-cad in preinvasive breast cancer, tissue samples from 200 patients with DCIS were analyzed, with 96 patients exhibiting a pure DCIS and 104 patients showing association of the DCIS with an invasive breast carcinoma. The median age of patients was 59 years (range 18–94 years). Informed consent was obtained from the patients, and the study was carried out in compliance with the Helsinki Declaration. The criteria proposed by Holland and coworkers [35], which consider nuclear grading and architectural differentiation (cellular polarization), were applied to classify all cases, resulting in gradings of low (*n *= 54), intermediate (*n *= 49) and high (*n *= 94). Based on these criteria, cases were finally classified as high-grade DCIS or non-high-grade DCIS (low and intermediate grade). All tissue samples were routinely paraformaldehyde fixed, paraffin embedded and archived at the Gerhard-Domagk Institute of Pathology, Münster University Hospital, Münster, Germany.

To allow permit simultaneous immunohistochemical staining of tissue samples using identical staining conditions, tissue microarrays (TMAs) were constructed from donor tissue blocks as described previously [36]. Following evaluation of haematoxylin and eosin stained microscope slides, at least four morphologically representative regions of each donor block were selected, and a TMA (2 cm × 3.5 cm) containing four to eight core specimens for each DCIS patient was assembled with a manual tissue arrayer (Beecher Instruments, Silver Springs, MD, USA) equipped with a 0.6 mm punch needle. The presence of DCIS in the TMA samples was verified on haematoxylin and eosin stained sections by a pathologist. Lobular carcinoma *in situ *(LCIS), defined by its distinctive histological appearance (characterized by small cells of low nuclear grade that fill and expand lobules without penetration of the basement membrane), was not included in the DCIS TMA. In cases of DCIS with a coexistent invasive carcinoma, only DCIS was included in the TMA sections because the invasive component was not available for analysis.

### Immunohistochemistry of TMAs

Consecutive 3 μm sections were cut from the TMA blocks and placed on poly-L-lysine coated coverslips. The dried coverslips were subsequently de-parrafinized, rehydrated and subjected to antigen retrieval by autoclaving in citrate buffer (pH 6.0; DAKO) or microwave treatment, as previously described [29]. After blocking of nonspecific binding, the sections were incubated with the following primary antibodies (specific for human target proteins): mouse anti-syndecan-1 (clone BB4, Serotec; 1:100), rabbit anti-c-met (Santa Cruz; 1:100), mouse anti-E-cadherin (BD Transduction Laboratories; 1:100), rabbit anti-VEGF-A (Santa Cruz; 1:15), rabbit-anti-VEGF-C (Santa Cruz; 1:25), rabbit anti-Flt-1 (Santa Cruz; 1:400), rabbit anti-KDR (Santa Cruz; 1:400), rabbit anti-Flt-4 (Santa Cruz; 1:400), rabbit anti-bFGF (basic FGF; Santa Cruz; 1:2000), rabbit anti-FGFR1 (Santa Cruz; 1:400), mouse-anti-ET-1 (endothelin-1; Affinity Bioreagents, Golden, CO, USA; 1:500), sheep anti-ET_A_R (ETAR; Alexis, Lausen, Switzerland; 1:800), sheep anti-ET_B_R (Alexis; 1:800). Oestrogen receptor (ER), progesterone receptor (PR) and human epidermal growth factor receptor (HER)2 were detected using standard immunohistochemical methods, as described previously [37]. As negative controls, the first antibody was either omitted or isotype-specific control IgGs (monoclonal antibodies) or nonimmune rabbit IgG (polyclonal antibodies) diluted to the same concentration as the first antibody were used. Following washing steps with PBS (three times for 5 min), the primary antibodies were detected using either the DAKO EnVision system (rabbit/mouse) with the Nova Red (Vector Labs) substrate (Sdc1, c-met and E-cad), APAAP (bFGF, Flt-1, KDR and FGFR1) or a labelled streptavidin-horseradish peroxidase method. For more details on retrieval techniques and incubations, the reader is referred to the report by Wülfing and coworkers [29].

### Slide scoring and statistical analysis

Semiquantitative analysis of staining results of 902 individual tissue array cores was performed by two investigators in a blinded manner without knowledge of the clinical data for the corresponding cases. Only core specimens that were confirmed to contain DCIS were included in the analysis. Consequently, not all cases could be analyzed for each individual marker. Sdc1, c-met and E-cad exhibited predominantly epithelial staining, which for the most part localized to the cell membrane. Additional cytoplasmic staining was frequently observed (for example, see Figure 3). Intensity of the immunostaining was scored semiquantitatively on an arbitrary four-tiered scale (negative = 0, weak = 1+, moderate = 2+ and strong = 3+). Grade 0 represented cases with no detectable immunostaining of tumour cells, whereas cases graded as 1+ exhibited weak staining of the majority of tumour cells. Tissue sections with a moderate or strong staining intensity were scored as 2+ or 3+, respectively. In the final score, samples with a moderate or strong immunostaining (score 2+ and 3+) were defined as 'positive', and samples with a weak or absent immunostaining as 'negative'.

An analogous scoring system was used to evaluate FGFR1, ET-1, ET_A_R and ET_B_R (cytoplasmic immunostaining), VEGF-C and Flt-4 (cytoplasmic and nuclear immunostaining), and KDR expression (cytoplasmic with occasional membraneous staining) [36]. Similarly, HER2 staining was scored on a scale ranging from 0 (absent) to 3+ (maximum cytomembranous staining), with a score above 2+ considered HER2 positive. The cytoplasmic immunostaining intensity of Flt-1 was classified on a three-tiered scale from 0 (negative) to 2+ (moderate), with cases scored as 1+ and 2+ considered Flt-1 'positive'. Expression of bFGF and VEGF-A was characterized as a negative or positive reaction according to both the percentage of stained tumour cells and the immunostaining intensity. When more than 10% of the tumour cells exhibited moderate or strong cytoplasmic immunoreaction, samples were defined as 'positive' [31]. ER and PR status were defined 'positive' when more than 10% nuclei stained positively.

Staining results for c-met, Sdc1 and E-cad were correlated with each other and with the expression of angiogenic and lymphangiogenic markers, as well as with ER, PR and HER2 status. In addition, expression of c-met, Sdc1 and E-cad was correlated with the type of DCIS (pure versus coexistent) and nuclear grading. Correlations were tested for statistical significance by cross tables, applying Pearson's χ^2 ^test and Fishers' exact test using SPSS version 10.0 for Windows (SPSS Inc., Chicago, IL, USA). The Bonferroni adjustment was calculated as α_adj _= 1 - (1 - α)^1/*n*^, with *n *= 17 tests [38].

## Results

### Colocalization and expression of Sdc1, E-cad and c-met in human breast cancer cell lines

**Figure 1:**
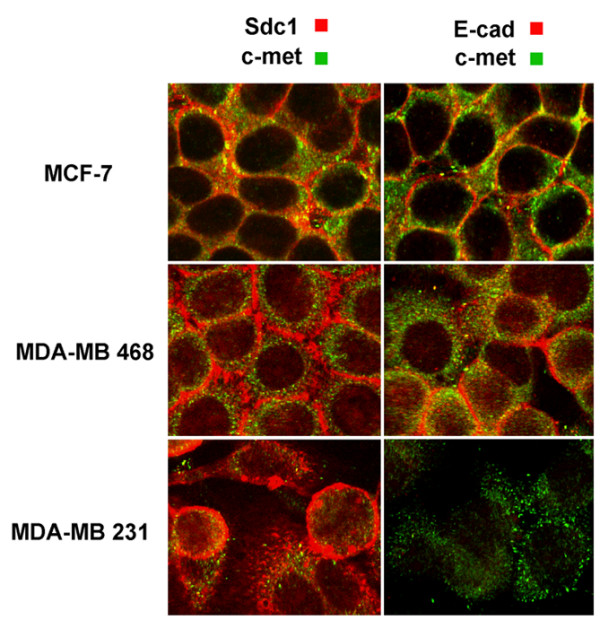
Immunolocalization of Sdc1, E-cad and c-met in human breast cancer cell lines. The human breast cancer cell lines MCF-7, MDA-MB468 and MDA-MB231 were stained with antibodies specific for c-met (green fluorescent secondary antibody) and Sdc1 or E-cad (red secondary antibodies), and observed by confocal immunofluorescence microscopy. Yellow staining denotes colocalization of c-met with Sdc1 or E-cad. E-cad, E-cadherin; Sdc, syndecan.

To characterize the interrelationship between the molecular markers investigated in this study, we performed confocal immunofluorescence microscopy colocalization experiments of c-met with Sdc1 and E-cad in human breast cancer cell lines. MCF-7, MDA-MB468 and MDA-MB231 cells, representing breast cancer cell lines of increasing dedifferentiation and metastatic capacity [39], were stained with antibodies specific for c-met, Sdc1 and E-cad (Figure 1). Sdc1 immunoreactivity was primarily located at the cell surface. In MDA-MB468 cells, a fuzzy membranous staining pattern of Sdc1 was observed, possibly indicating alterations in cell-cell contacts. Similar to Sdc1, E-cad staining localized to the plasma membrane. However, an inverse correlation of E-cad expression with the degree of tumour cell de-differentiation was observed, because membraneous staining was almost absent in MDA-MB231 cells. A punctate staining pattern was exhibited by c-met. In MCF-7 cells, c-met partially colocalized with Sdc1 and E-cad. The degree of colocalization appeared to be higher in MCF-7 cells than in MDA-MB468 cells. In MDA-MB231 cells, colocalization with c-met could not be assessed because of the low level of E-cad expression.

**Figure 2:**
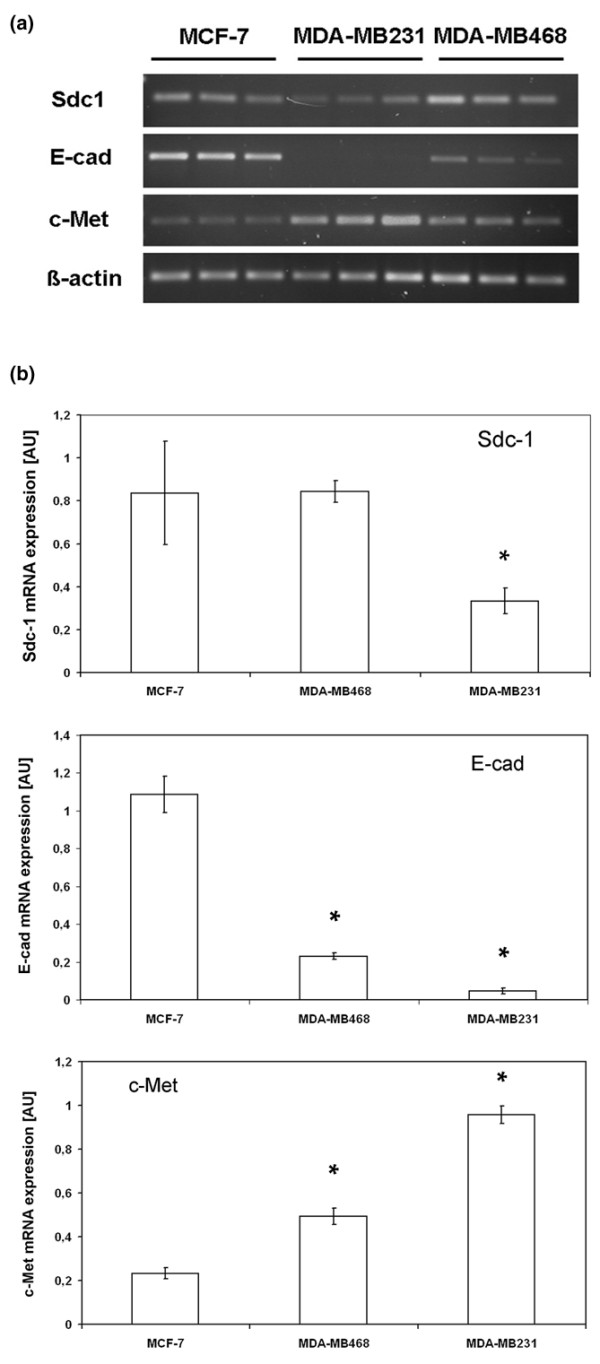
RT-PCR analysis of Sdc1, E-cad and c-met in human breast cancer cell lines. RNA was prepared from MCF-7, MDA-MB468 and MDA-MB231 breast cancer cells; mRNA was reverse transcribed and used as a template for PCR amplification of Sdc1, E-cad and c-met (as described in Materials and methods section). **(a) **PCR band intensities were normalized for actin expression and the data from three independent experiments were analyzed using the paired Student's *t*-test. (b) *P < 0.01 (Sdc1: MDA-MB468 versus MDA-MB231; E-cad and c-met: MCF-7 versus MDA-MB231, MCF-7 versus MDA-MB468 and MDA-MB468 versus MDA-MB231). AU, arbitrary units; E-cad, E-cadherin; RT-PCR, reverse transcription polymerase chain reaction; Sdc, syndecan.

We next compared expression of Sdc1, E-cad and c-met at the mRNA level by semiquantitative RT-PCR (Figure 2). Similar levels of Sdc1 expression were observed in MCF-7 and MDA-MB468 cells, but Sdc1 mRNA expression was significantly lower (*P *< 0.001) in MDA-MB231 cells than in MDA-MB468 cells. With increasing de-differentiation and higher metastatic potential (MCF-7 < MDA-MB468 < MDA-MB231), a significant decrease in E-cad mRNA expression was observed (Figure 2b). Of note, expression levels of c-met exhibited an inverse relation, with the highest expression in MDA-MB231 cells (Figure 2).

### Immunohistochemical staining of Sdc1, E-cad and c-met in DCIS

**Figure 3:**
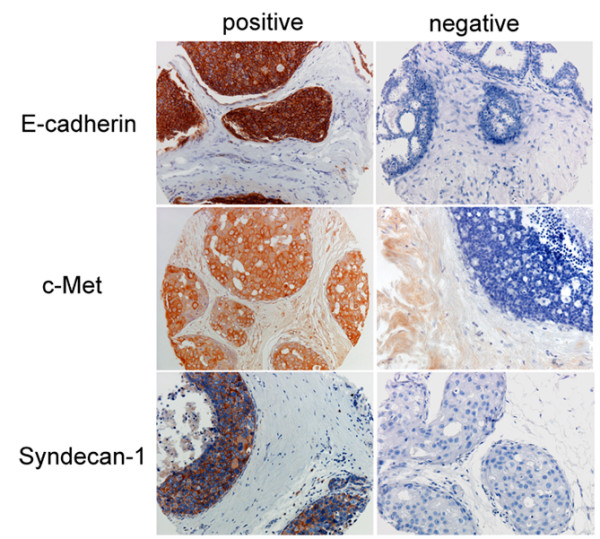
Representative immunohistochemical staining patterns for E-cad, c-met, and Sdc1 in DCIS. Examples for presence (positive) and absence (negative) of marker expression are shown. DCIS, ductal carcinoma *in situ*; E-cad, E-cadherin; Sdc, syndecan.

We next employed TMA technology to determine the expression of Sdc1, E-cad and c-met in 200 cases of DCIS of the breast. Tumour cells exhibited variable expression of Sdc1, ranging from weak to strong. Staining was predominantly membranous but often additionally cytoplasmic. Stromal cells and myoepithelial cells showed no immunoreactivity (Figure 3). For c-met there was weak to strong membrane bound and additional cytoplasmic expression in stromal cells, and myoepithelial cells remained negative (Figure 3). Expression of E-cadherin was observed in almost all cases of DCIS, with the majority exhibiting strong immunoreactivity. Staining in tumour cells was membranous and cytoplasmic, whereas stroma and myoepithelium remained negative (Figure 3).

Detailed staining results for all three markers in DCIS are listed in Table 2. Based on a four-tiered scale, moderate to strong positive staining of tumour cells was observed for Sdc1 in 108 out of 150 (72%), for c-met in 69 out of 142 (48.6%) and for E-cad in 101 out of 149 (67.8%) evaluable DCIS samples. We next investigated the interrelation of Sdc1, c-met and E-cad expression. Expression of E-cad was significantly correlated with expression of c-met (*P *= 0.004) and Sdc1 (*P *= 0.007; Table 3).

### Correlation of Sdc1, c-met and E-cad expression with histopathological characteristics

A significant correlation was observed between expression of c-met and HER2 (*P *= 0.044), and there was a trend toward an association of HER2 with Sdc1 expression (*P *= 0.057; Table 3). No significant correlation of high-to-moderate c-met, Sdc-1 and E-cad expression with ER and PR status or high-grade DCIS [35] was observed (Table 3). Expression of c-met (*P *< 0.001) and Sdc1 (*P *= 0.037) was significantly more frequent in the subgroup of pure DCIS than in the subgroup of DCIS with a coexistent invasive carcinoma (Table 4). These differences were not due to an unequal distribution of histopathologic or clinical features, because analysis of the distribution of patients' age, ER, PR and HER2 status, and nuclear grading revealed no statistically significant differences between three subgroups (data not shown).

### Correlation of c-met, Sdc1 and E-cad expression with angiogenic and lymphangiogenic factors

Expression of four ligand-receptor pairs known to play a role in angiogenesis and lymphangiogenesis was analyzed to test for a potential association of c-met, Sdc1 and E-cad expression with such angiogenic and lymphangiogenic factors: VEGF-A and its receptors Flt-1 and KDR; bFGF and its receptor FGFR1; the endothelin axis, comprised of ET-1 and its receptors ET_A_R and ET_B_R; and the lymphangiogenic factor VEGF-C and its receptor Flt-4 (Table 5).

VEGF-A exhibited faint to moderate cytoplasmic staining of tumour cells. Positive staining was also observed in myoepithelial cells, whereas normal glandular epithelial cells as well as stromal and connective tissue were mostly VEGF-A negative. KDR staining presented as strong cytoplasmic staining. Normal glandular epithelial cells stained positive for KDR, whereas stroma and connective tissue exhibited no staining. Flt-1 immunoreactivity presented as a granular cytoplasmic staining of tumour cells and of the peritumoural stroma. Normal glandular epithelial cells stained negative for Flt-1.

bFGF staining of tumour cells was predominantly nuclear and occasionally cytoplasmic. Although the stroma was bFGF negative, positive staining of normal glandular epithelial cells was frequently observed. Tumor cells exhibited strong cytoplasmic staining for FGFR1, whereas normal glandular cells were moderately positive, and the majority of stromal cells were weakly positive.

Expression of ET-1, ET_A_R and ET_B_R presented as positive cytoplasmic staining of tumour cells, and a faint additional stromal staining was observed in some ET-1 positive cases. Normal glandular epithelial cells were mostly negative for markers of the endothelin axis.

VEGF-C staining presented as strong cytoplasmic staining of tumour cells, whereas normal breast epithelium was largely VEGF-C negative, as was the stroma and connective tissue. Tumor cells stained strongly positive for Flt-4, and positive staining was also frequently observed in normal glandular epithelium. In contrast, no Flt-4 staining was detected in the stroma and connective tissues.

A highly significant correlation of c-met expression with ET_A_R and ET_B_R was found; 92.6% of c-met positive DCIS versus 56.7% of c-met negative DCIS stained positive for ET_A_R (*P *< 0.001), and 57.4% of c-met positive tumours versus 23.4% of c-met negative tumours stained positive for ET_B_R (*P *< 0.001). Furthermore, c-met expression exhibited a significant positive correlation with expression of VEGF-A (*P *= 0.040) and FGFR1 (*P *= 0.028; Table 5).

Similar to c-met, E-cad expression was significantly correlated with ET_A_R expression (*P *< 0.001). In addition, we observed a significant correlation of E-cad expression with positive staining for VEGF-A (*P *= 0.005) and VEGF-C (*P *= 0.048).

Sdc1 exhibited a trend toward correlation with lymphangiogenic factors (VEGF-C, *P *= 0.061; Flt-4, *P *= 0.071; Table 5).

## Discussion

In this study we characterized coexpression of the functionally linked prognostic markers Sdc1, E-cad and c-met in 200 DCIS using TMA technology and in different human breast cancer cell lines. Our initial objective was to identify a group of functionally interrelated markers that have prognostic significance in breast cancer. In this regard we observed significant correlations between expression levels for the markers c-met, Sdc1 and E-cad in DCIS. The markers c-met and Sdc1 were significantly more frequently expressed in pure DCIS than in DCIS with a coexistent invasive carcinoma. In the case of Sdc1, this finding may indicate downregulation during epithelial-mesenchymal transition [40,41], which has been demonstrated in mammary epithelial cells *in vitro *[23,24] and during development [22]. Regarding the association between the expression pattern of Sdc1, E-cad and c-met with disease progression, future studies could investigate the correlation with expression for coexisting DCIS and invasive carcinoma. However, based on our cell culture data and on current knowledge on the prognostic value and biological function of the markers (see Table 1), we speculate that a reduction in E-cad expression and an upregulation of Sdc1 expression may occur in the invasive component of mixed lesions. In the case of c-met, our finding that c-met expression is highest in MDA-MB231 cells, and exhibits a positive correlation with other features of aggressiveness such as HER2 overexpression seemingly contradicts our observation that c-met expression is higher in pure DCIS than in DCIS associated with invasive cancer. We recently observed significantly increased expression of prognostically unfavourable proangiogenic factors in pure DCIS compared with coexistent DCIS [29]. This is consistent with findings reported by Teo and coworkers [42], who described different vascular density and phenotypes in pure versus coexistent DCIS. Therefore, the preferential association of c-met expression with pure DCIS may be indicative of the increased angiogenic activity in pure DCIS [11,29].

The metastatic origin of the investigated human breast cancer cell lines [39] has inherent limitations with respect to a direct comparison of marker expression in DCIS tissue. However, the positive correlation of c-met, Sdc1 and E-cad in an early stage of tumour progression at the tissue level was largely supported by our colocalization studies in these cell lines, in which partial colocalization was observed between c-met and Sdc1 and E-cad, respectively, in the less aggressive MCF-7 and MDA-MB468 cell lines. In addition, Sdc1 and E-cad exhibited a similar cell surface distribution in these cells. Moreover, progressive loss of E-cad expression and a progressive increase in c-met expression were observed with increasing de-differentiation and higher metastatic potential (MCF-7 < MDA-MB468 < MDA-MB231) of the breast cancer cell lines at the mRNA level.

These findings are similar to the expression patterns of E-cad and c-met observed in clinical studies. Reduced expression of E-cad in breast carcinoma is associated with shortened disease-free survival and high histological grade [43-46], whereas high expression of c-met is associated with more aggressive disease and decreased disease-free survival in node-negative breast cancer [15,47] (see Table 1). In addition, coordinated overexpression of c-met and the oncogene RON is observed in MBA-MB231 but not in MCF-7 cells [15]. Our observation of a synchronous downregulation of E-cad and Sdc1 mRNA in highly aggressive MDA-MB231 cells is in accordance with previous *in vitro *experiments on mammary epithelial cells [23,24]. Expression of Sdc1 can be regulated at the post-transcriptional level [48,49], which may explain the strong Sdc1 protein expression observed by confocal immunofluorescence microscopy in MDA-MB231 cells (Figure 1). The heterogeneity in Sdc1 and E-cad coexpression in breast cancer cell lines of different degrees of de-differentiation demonstrates that the concept of synchronous regulation of Sdc1 and E-cad in epithelial cells [23,24] cannot be fully adapted to breast cancer. Our data suggest that the degree of coexpression is higher in breast cells that exhibit a more benign phenotype, such as DCIS, whereas coexpression may be lost in more advanced stages of tumour progression. These findings could help to explain why no correlation was found between E-cad and Sdc1 expression in a patient collective containing ductal invasive, lobular and tubular breast cancer tissues [8], whereas a correlation was found in our more uniform DCIS collective.

We observed no significant association of c-met, E-cad and Sdc-1 expression with hormone receptor status in DCIS. In patient collectives including more advanced stages of breast cancer progression, clinical and experimental data suggest a possible link between loss of E-cad expression and ER-negative status [20,43,50]. Moreover, recent studies have linked Sdc1 expression to an aggressive, ER-negative breast carcinoma subtype [6-8]. In order to distinguish between low and high expression levels of possibly synchronously regulated Sdc1 and E-cad, both low expression and absence of staining for the markers investigated were scored as 'negative'. Although application of this scoring method was necessary to detect synchronous coregulation of marker expression, it might have lead to failure to demonstrate significant associations of E-cad expression with hormone receptor status and grade of DCIS in the present study. Although it may occasionally be difficult to distinguish between DCIS and LCIS in cases where LCIS may involve extralobular ducts, we can largely exclude the possibility that the analyzed DCIS cases were erroneously scored as LCIS (see Materials and methods, above).

We found a significant correlation between HER2 and c-met expression. This correlation may be of functional importance, because both molecules are tyrosine kinase receptors that promote cell proliferation [11,51]. Of note, Khoury and coworkers [51] recently demonstrated that HGF-mediated c-met signalling in the presence of constitutively active HER2 leads to a downregulation of E-cad and promotes epithelial-mesenchymal transition in MDCK cells. Thus, signalling downstream of the c-met receptor synergizes with HER2 to enhance a malignant phenotype. In a similar manner, additional receptors could contribute to this synergism in signalling, and our finding of c-met correlating with FGFR1 expression does support this view. Moreover, this hypothesis is supported by a study on the expression of c-met and the tyrosine kinase receptor RON in node-negative breast cancer patients [15]. Ten-year disease-free survival was significantly decreased in patients with tumours expressing only one of the two markers, and it was even worse in patients with RON-positive/c-met-positive tumours. Because Sdc1 is an established coreceptor for several receptor tyrosine kinases, including FGFR1 [1,3] and c-met [10], it could also contribute to synergistic signalling. We observed a trend toward a correlation of Sdc1 and HER2 expression in DCIS (*P *= 0.057). This is in accordance with the study conducted by Barbareschi and coworkers [6], which demonstrated positive correlation of Sdc1 and HER2 in a collective of 254 invasive breast carcinomas. In accordance with its role as a signalling coreceptor, a recent study conducted in 207 breast cancer patients demonstrated a significant association of Sdc1 overexpression with the Ki67 proliferation index [8].

In DCIS, an increase in periductal blood vessels has been correlated with recurrence of invasive disease [52], demonstrating the relevance of angiogenesis to tumour progression. Expression of the angiogenic factor VEGF-A in tumour cells from patients with DCIS was shown to correlate with the degree of angiogenesis [53]. In the present study, we found a significant correlation of both c-met and E-cad with VEGF-A expression in DCIS. In addition, c-met expression exhibited a negative correlation with Flt-1 expression; Flt-1 is a negative regulator of VEGF availability, which is regarded as an antiangiogenic receptor. Our findings constitute the first characterization of a proangiogenic role for c-met, Sdc1 and E-cad in DCIS, but they are in accordance with published work on the biological role of these molecules. Ribozyme targeting of c-met in mammary cancer cells reduced mammary cancer and tumour-associated angiogenesis in a xenograft model [12]. In addition, the HGF-antagonist NK4 inhibits c-met signalling and angiogenesis *in vitro *[32] and reduces intratumoural microvessel density in a hepatocellular carcinoma xenograft model [54]. Sdc1 acts as a coreceptor for several angiogenic and growth factors, including multiple VEGF isoforms and bFGF [1,3]. *In vivo*, we recently demonstrated increased corneal angiogenesis in Sdc-1 deficient mice and the formation of abnormally dilated blood vessels in skin wounds of Sdc-1 overexpressing mice, indicating a regulatory role for Sdc1 in angiogenesis [2,4]. Moreover, in a nude mouse xenograft model, coinjection of MDA-MB-231 breast cancer cells with Sdc-1 overexpressing and mock-transfected mouse fibroblasts resulted in significantly elevated microvessel density and a larger vessel area in tumours containing Sdc1 overexpressing stroma cells [33]. A TMA analysis of 207 human breast cancer samples by the same authors revealed that stromal Sdc1 expression correlated with vessel density and total vessel area, demonstrating a role for Sdc1 in breast tumour angiogenesis.

Although we observed a trend toward an association of Sdc1 expression with markers of lymphangiogenesis, we could not demonstrate a statistically significant correlation of Sdc1 expression and proangiogenic markers in DCIS (Table 5). This finding may suggest that the angiogenesis-associated functions of Sdc1 primarily contribute to more advanced stages of tumour progression. We have recently demonstrated that VEGF-C and Flt-4, which are established mediators of lymphangiogenesis [55,56], were expressed in the tumour cells of a large proportion (88% to 95%) of DCIS specimens [29]. In the present study, we found a significant association of E-cad with VEGF-C expression (*P *= 0.048). Althuogh very little is known about the role of E-cad in lymphangiogenesis, by virtue of its biological role an involvement in this process could easily be envisaged. Moreover, the correlations of VEGF-A/C with E-cad and of VEGF-A with c-met could be a sign of an angiogenic stimulation of the stroma by the tumour cells [57]. Furthermore, the preferential expression of Sdc1 and c-met in pure versus coexistent DCIS parallels the significantly more common expression of proangiogenic factors in pure versus coexistent DCIS [29].

ET-1 and its G-protein-coupled receptors ET_A_R and ET_B_R (the endothelin axis) are overexpressed in breast carcinomas, and influence angiogenesis, tumourigenesis and tumour progression [34,36,58]. Moreover, we previously demonstrated overexpression of the ET axis in DCIS and observed an association with different angiogenic and lymphangiogenic factors [29]. An important novel finding of the present study is the highly significant association of c-met and E-cad expression with ET receptor expression. Similar to ET_A_R [34], c-met expression is upregulated by hypoxia [59], thus promoting invasive growth of tumour cells. In addition, the c-met ligand HGF has been shown to inhibit ET-1 release in endothelial cells, indicating a possible link between c-met mediated signalling and the ET axis [60]. However, the coexpression of c-met and the ET receptors may simply indicate an upregulation of parallel signalling pathways that utilize different upstream ligands and converge intracellularly. Sdc1 and E-cad have been associated with epithelial-mesenchymal transition [22,40], and it has recently been shown that ET-1 mediated signalling is required during epithelial-mesenchymal transition in ovarian cancer progression [61,62]. In ovarian cancer [61,62] and melanoma [63,64], activation of the ET axis results in downregulated E-cad expression. In DCIS, coexpression of ET receptors, E-cad, c-met and Sdc1 may constitute a specific expression signature indicative of the transition of an early stage to later stages of tumour progression.

One possible caveat associated with the present study is the large number of statistical tests that were performed (*n *= 17). Although the statistical analysis employed in this study permits easier comparison and interpretation of *P *values within the context of the results from other studies, the reader must keep in mind that some significant associations may be falsely positive. Using a Bonferroni adjustment, the corrected significance level for the tests employed in this study would be *P *< 0.003. However, although corrections for multiple comparisons on the one hand reduce the chances of making a type I error, the chances of making a type II error are increased. Moreover, the relevance of the null hypothesis underlying Bonferroni adjustments has recently been questioned (see the report by Perneger [38] for a discussion). Therefore, there remains a lack of consensus within the scientific community regarding whether corrections for multiple comparisons are applicable.

## Conclusion

In the present study we demonstrated coexpression of c-met, Sdc1 and E-cad in DCIS. Of note, expression of c-met, Sdc1 and E-cad correlated with expression of angiogenic and lymphangiogenic factors, including endothelin receptor expression. It is tempting to speculate that this expression signature indicates a state of a parallel, synergistic activation of multiple receptor pathways (receptor tyrosine kinases, G-protein-coupled receptors and nuclear transcription factors), which vastly promote tumour cell proliferation and angiogenesis in the tumour stroma. Since these activation pathways rely on different molecular mechanisms, therapeutic approaches must take multiple targets into consideration. Inhibitors of tyrosine kinases, of the endothelin axis, of heparan sulphate-modulated signalling and of angiogenesis are currently being evaluated in clinical trials or have already found their way into cancer therapies [5,65-69]. Data from the present study may help in the development of more efficient therapeutic approaches in breast cancer, addressing the need to target simultaneously multiple molecules that are relevant to this early step of breast cancer progression.

## Abbreviations

bFGF = basic fibroblast growth factor; DCIS = ductal breast carcinoma *in situ*; E-cad = E-cadherin; ER = oestrogen receptor; ET = endothelin; ET_A/B_R = endothelin A/B receptor; FGFR = fibroblast growth factor receptor; HER = human epidermal growth factor receptor; HGF = hepatocyte growth factor; PBS = phosphate-buffered saline; PR = progesterone receptor; RT-PCR = reverse transcription polymerase chain reaction; Sdc1 = syndecan-1; TMA = tissue microarray; VEGF = vascular endothelial growth factor.

## Competing interests

The authors declare that they have no competing interests.

## Authors' contributions

MG participated in the design and coordination of the study, participated in TMA slide analysis, performed the cell culture studies and drafted the manuscript. CK performed construction of the TMA and analysis of staining results. IR participated in statistical analysis and helped to draft the manuscript. LK provided general support, participated in study design and helped to draft the manuscript. PW participated in the design and coordination of the study, performed the statistical analysis and drafted the manuscript.

## Figures and Tables

**Table 1:** Cancer-related functions and interrelation of Sdc1, c-met and E-cad

Marker	Molecular characteristics	Biological functions relevant to cancer^a^	Clinical relevance to breast cancer^a^	Relation to c-met^a^	Relation to E-cad^a^
Sdc1	Cell surface heparan sulphate proteoglycan	Cell and matrix receptor [1] Coreceptor for chemokines, angiogenic and growth factors, and modulator of proteolytic activity [2-4,16,57]	Positive correlation with poor prognosis and tumour angiogenesis [6-8,33] Predictive factor in neoadjuvant chemotherapy [9]	Sdc1 is c-met co-receptor in multiple myeloma [10]	Coordinated regulation and codistribution in mammary tumor cells and epithelial-mesenchymal transition [22-25] β-Catenin responsive progenitor cells depend on Sdc1 [27,28]
c-met	Transmembrane tyrosine kinase receptor for hepatocyte growth factor	c-met pathway modulates cell dissociation and motility, protease overexpression and stimulates angiogenesis [11,12]	Expression associated with poor outcome in patients with (axillary) lymph node negative breast cancer [15,47,70] Negative prognostic factor in breast cancer [71,72]	Not applicable	Correlation with abnormal β-catenin expression suggests downregulation of E-cad/β-catenin by c-met [73]
E-cad	Epithelial calcium-dependent cell adhesion molecule	Ensures structural integrity, and contact inhibition of epithelia [21] Expression changes during epithelial-mesenchymal transition [40] Involved in β-catenin-mediated signalling [20]	Membranous staining is independent predictor for disease-free survival in lobular breast cancer [44] Loss of expression is associated with negative ER status, high histological grade, metastasis and poor prognosis in breast cancer [43,45,46,74,75] E-cad is used to distinguish ductal from lobular neopasia [18,19]	(See Sdc1)	Not applicable

^a^Selected examples are given, along with references. E-cad, E-cadherin; ER, oestrogen receptor; Sdc, syndecan.

**Table 2:** Immunohistochemical analysis of Sdc1, c-met and E-cad expression in DCIS of the breast

Score	c-met (*n *= 142)	E-cad (*n *= 149)	Sdc1 (*n *= 150)
0	10 (7)	11 (7.4)	4 (2.7)
1	63 (44.4)	37 (24.8)	38 (25.3)
2	51 (35.9)	89 (59.7)	103 (68.7)
3	18 (12.7)	12 (8.1)	5 (3.3)
Negative (score 0–1)	73(51.4)	48 (32.2)	42 (28)
Positive (score 2–3)	69 (48.6)	101 (67.8)	108 (72)

Values are expressed as number of evaluable tissue specimens (%). DCIS, ductal carcinoma *in situ*; E-cad, E-cadherin; Sdc, syndecan.

**Table 3:** Interrelation of c-met, E-cad and Sdc1 expression in DCIS and correlation with histopathological characteristics

	c-met			E-cad			Sdc1		
	
	Negative (*n *= 73)	Positive (*n *= 69)	*P*	Negative (*n *= 48)	Positive (*n *= 101)	*P*	Negative (*n *= 42)	Positive (*n *= 105)	*P*
c-met positive	0.0%	100.0%		30.8%	58.8%	0.004	38.2%	56.3%	0.071
E-cad positive	56.5%	80.6%	0.004	0.0%	100.0%		51.4%	76.0%	0.007
Sdc1 positive	66.7%	80.6%	0.071	58.5%	80.9%	0.007	0.0%	100.0%	
High grade DCIS	47.2%	48.5%	0.877	47.9%	47.5%	0.960	37.5%	49.5%	0.193
ER positive	25.4%	31.3%	0.463	20.0%	31.5%	0.176	37.1%	26.0%	0.251
PR positive	33.9%	37.9%	0.643	37.8%	33.7%	0.655	40.0%	31.6%	0.368
ErbB2 positive	12.5%	27.3%	0.044	23.5%	23.9%	0.964	11.8%	28.0%	0.057

DCIS, ductal carcinoma *in situ*; E-cad, E-cadherin; ER, oestrogen receptor; PR, progesterone receptor; Sdc, syndecan.

**Table 4:** Expression of c-met, Sdc1 and E-cad in the subgroups of pure DCIS and DCIS with coexistent invasive carcinoma

Molecular marker	Pure DCIS	Coexistent DCIS	*P*
Sdc1	59/74 (79.7%)	49/76 (64.5%)	0.037
c-met	47/72 (65.3%)	22/70 (31.4%)	<0.001
E-cad	56/76 (73.7%)	45/73 (61.6%)	0.116

DCIS, ductal carcinoma *in situ*; E-cad, E-cadherin; Sdc, syndecan.

**Table 5:** Correlation of c-met, Sdc1 and E-cad expression with angiogenic and lymphangiogenic factors

	c-met			E-cad			Sdc1		
	
	Negative (*n *= 73)	Positive (*n *= 69)	*P*	Negative (*n *= 48)	Positive (*n *= 101)	*P*	Negative (*n *= 42)	Positive (*n *= 105)	*P*
VEGF-A	36.2%	53.8%	0.040	26.2%	52.0%	0.005	55%	41.6%	0.149
Flt-1	18.6%	7.7%	0.063	14.3%	11.5%	0.642	12.8%	12.5%	0.959
KDR	48.4%	63.1%	0.094	47.4%	56.4%	0.347	45.7%	58.3%	0.198
VEGF-C	84.5%	94.1%	0.068	80.4%	91.8%	0.048	80.5%	91.5%	0.061
Flt-4	92.9%	98.6%	0.099	95.1%	97.0%	0.584	90.0%	97.2%	0.071
ET-1	47.7%	59.7%	0.176	52.4%	48.9%	0.709	47.4%	51.6%	0.661
ET_A_R	56.7%	92.6%	<0.001	53.8%	86.9%	<0.001	70.3%	79.0%	0.277
ET_B_R	23.4%	57.4%	<0.001	30.8%	43.3%	0.177	31.6%	47.0%	0.102
BFGF	17.4%	11.9%	0.394	10.0%	13.4%	0.583	18.9%	11.4%	0.250
FGFR1	89.1%	98.4%	0.028	92.3%	95.9%	0.394	91.4%	96.0%	0.287

bFGF, basic fibroblast growth factor; E-cad, E-cadherin; ET, endothelin; ET_A/B_R, endothelin A/B receptor; FGFR, fibroblast growth factor receptor; Sdc, syndecan; VEGF, vascular endothelial growth factor.

## References

[B1] Bernfield M, Götte M, Park PW, Reizes O, Fitzgerald ML, Lincecum J, Zako M (1999). Functions of cell surface heparan sulfate proteoglycans. Annu Rev Biochem.

[B2] Götte M, Joussen AM, Klein C, Andre P, Wagner DD, Hinkes MT, Kirchhof B, Adamis AP, Bernfield M (2002). Role of syndecan-1 in leukocyte-endothelial interactions in the ocular vasculature. Invest Ophthalmol Vis Sci.

[B3] Götte M (2003). Syndecans in inflammation. FASEB J.

[B4] Elenius V, Götte M, Reizes O, Elenius K, Bernfield M (2004). Inhibition by the soluble syndecan-1 ectodomains delays wound repair in mice overexpressing syndecan-1. J Biol Chem.

[B5] Yip GW, Smollich M, Götte M (2006). Therapeutic value of glycosaminoglycans in cancer. Mol Cancer Ther.

[B6] Barbareschi M, Maisonneuve P, Aldovini D, Cangi MG, Pecciarini L, Angelo Mauri F, Veronese S, Caffo O, Lucenti A, Palma PD (2003). High syndecan-1 expression in breast carcinoma is related to an aggressive phenotype and to poorer prognosis. Cancer.

[B7] Leivonen M, Lundin J, Nordling S, von Boguslawski K, Haglund C (2004). Prognostic value of syndecan-1 expression in breast cancer. Oncology.

[B8] Baba F, Swartz K, van Buren R, Eickhoff J, Zhang Y, Wolberg W, Friedl A (2006). Syndecan-1 and syndecan-4 are overexpressed in an estrogen receptor-negative, highly proliferative breast carcinoma subtype. Breast Cancer Res Treat.

[B9] Götte M, Kersting C, Ruggiero M, Tio J, Tulusan AH, Kiesel L, Wülfing P (2006). Predictive value of syndecan-1 expression for the response to neoadjuvant chemotherapy of primary breast cancer. Anticancer Res.

[B10] Derksen PW, Keehnen RM, Evers LM, van Oers MH, Spaargaren M, Pals ST (2002). Cell surface proteoglycan syndecan-1 mediates hepatocyte growth factor binding and promotes Met signaling in multiple myeloma. Blood.

[B11] Peruzzi B, Bottaro DP (2006). Targeting the c-met signaling pathway in cancer. Clin Cancer Res.

[B12] Jiang WG, Grimshaw D, Martin TA, Davies G, Parr C, Watkins G, Lane J, Abounader R, Laterra J, Mansel RE (2003). Reduction of stromal fibroblast-induced mammary tumor growth, by retroviral ribozyme transgenes to hepatocyte growth factor/scatter factor and its receptor, c-MET. Clin Cancer Res.

[B13] Bertotti A, Comoglio PM, Trusolino L (2005). Beta4 integrin is a transforming molecule that unleashes Met tyrosine kinase tumorigenesis. Cancer Res.

[B14] Welm AL, Kim S, Welm BE, Bishop JM (2005). MET and MYC cooperate in mammary tumorigenesis. Proc Natl Acad Sci USA.

[B15] Lee WY, Chen HH, Chow NH, Su WC, Lin PW, Guo HR (2005). Prognostic significance of co-expression of RON and MET receptors in node-negative breast cancer patients. Clin Cancer Res.

[B16] Beauvais DM, Rapraeger AC (2004). Syndecans in tumor cell adhesion and signaling. Reprod Biol Endocrinol.

[B17] Beauvais DM, Burbach BJ, Rapraeger AC (2004). The syndecan-1 ectodomain regulates alphavbeta3 integrin activity in human mammary carcinoma cells. J Cell Biol.

[B18] Lerwill MF (2004). Current practical applications of diagnostic immunohistochemistry in breast pathology. Am J Surg Pathol.

[B19] Acs G, Lawton TJ, Rebbeck TR, LiVolsi VA, Zhang PJ (2001). Differential expression of E-cadherin in lobular and ductal neoplasms of the breast and its biologic and diagnostic implications. Am J Clin Pathol.

[B20] Cowin P, Rowlands TM, Hatsell SJ (2005). Cadherins and catenins in breast cancer. Curr Opin Cell Biol.

[B21] Cavallaro U, Liebner S, Dejana E (2006). Endothelial cadherins and tumor angiogenesis. Exp Cell Res.

[B22] Sun D, Mcalmon KR, Davies JA, Bernfield M, Hay ED (1998). Simultaneous loss of expression of syndecan-1 and E-cadherin in the embryonic palate during epithelial-mesenchymal transformation. Int J Dev Biol.

[B23] Kato M, Saunders S, Nguyen H, Bernfield M (1995). Loss of cell surface syndecan-1 causes epithelia to transform into anchorage-independent mesenchyme-like cells. Mol Biol Cell.

[B24] Leppä S, Vleminckx K, Van Roy F, Jalkanen M (1996). Syndecan-1 expression in mammary epithelial tumor cells is E-cadherin-dependent. J Cell Sci.

[B25] Zimmermann P, Tomatis D, Rosas M, Grootjans J, Leenaerts I, Degeest G, Reekmans G, Coomans C, David G (2001). Characterization of syntenin, a syndecan-binding PDZ protein, as a component of cell adhesion sites and microfilaments. Mol Biol Cell.

[B26] Alexander CM, Reichsman F, Hinkes MT, Lincecum J, Becker KA, Cumberledge S, Bernfield M (2000). Syndecan-1 is required for Wnt-1-induced mammary tumorigenesis in mice. Nat Genet.

[B27] Liu BY, Kim YC, Leatherberry V, Cowin P, Alexander CM (2003). Mammary gland development requires syndecan-1 to create a beta-catenin/TCF-responsive mammary epithelial subpopulation. Oncogene.

[B28] Liu BY, McDermott SP, Khwaja SS, Alexander CM (2004). The transforming activity of Wnt effectors correlates with their ability to induce the accumulation of mammary progenitor cells. Proc Natl Acad Sci USA.

[B29] Wülfing P, Kersting C, Buerger H, Mattsson B, Mesters R, Gustmann C, Hinrichs B, Tio J, Böcker W, Kiesel L (2005). Expression patterns of angiogenic and lymphangiogenic factors in ductal breast carcinoma in situ. Br J Cancer.

[B30] Wu Y, Hooper AT, Zhong Z, Witte L, Bohlen P, Rafii S, Hicklin DJ (2006). The vascular endothelial growth factor receptor (VEGFR-1) supports growth and survival of human breast carcinoma. Int J Cancer.

[B31] De Jong JS, van Diest PJ, van der Valk P, Baak JP (1998). Expression of growth factors, growth inhibiting factors, and their receptors in invasive breast cancer. I: an inventory in search of autocrine and paracrine loops. J Pathol.

[B32] Jiang WG, Hiscox SE, Parr C, Martin TA, Matsumoto K, Nakamura T, Mansel RE (1999). Antagonistic effect of NK4, a novel hepatocyte growth factor variant, on in vitro angiogenesis of human vascular endothelial cells. Clin Cancer Res.

[B33] Maeda T, Desouky J, Friedl A (2006). Syndecan-1 expression by stromal fibroblasts promotes breast carcinoma growth in vivo and stimulates tumor angiogenesis. Oncogene.

[B34] Wülfing P, Götte M, Sonntag B, Kersting C, Schmidt H, Wülfing C, Buerger H, Greb R, Böcker W, Kiesel L (2005). Overexpression of Endothelin-A-receptor in breast cancer: regulation by estradiol and cobalt-chloride induced hypoxia. Int J Oncol.

[B35] Holland R, Peterse JL, Millis RR, Eusebi V, Faverly D, van de Vijver MJ, Zafrani B (1994). Ductal carcinoma in situ: a proposal for a new classification. Semin Diagn Pathol.

[B36] Wülfing P, Kersting C, Tio J, Fischer RJ, Wülfing C, Poremba C, Diallo R, Böcker W, Kiesel L (2004). Endothelin-1-, endothelin-A-, and endothelin-B-receptor expression is correlated with vascular endothelial growth factor expression and angiogenesis in breast cancer. Clin Cancer Res.

[B37] Wülfing P, Diallo R, Müller C, Wülfing C, Poremba C, Heinecke A, Rody A, Greb RR, Böcker W, Kiesel L (2003). Analysis of cyclooxygenase-2 expression in human breast cancer: high throughput tissue microarray analysis. J Cancer Res Clin Oncol.

[B38] Perneger TV (1998). What's wrong with Bonferroni adjustments. BMJ.

[B39] Lacroix M, Leclercq G (2004). Relevance of breast cancer cell lines as models for breast tumours: an update. Breast Cancer Res Treat.

[B40] Lombaerts M, van Wezel T, Philippo K, Dierssen JW, Zimmerman RM, Oosting J, van Eijk R, Eilers PH, van de Water B, Cornelisse CJ (2006). E-cadherin transcriptional downregulation by promoter methylation but not mutation is related to epithelial-to-mesenchymal transition in breast cancer cell lines. Br J Cancer.

[B41] Vos CB, Cleton-Jansen AM, Berx G, de Leeuw WJ, ter Haar NT, van Roy F, Cornelisse CJ, Peterse JL, van de Vijver MJ (1997). E-cadherin inactivation in lobular carcinoma in situ of the breast: an early event in tumorigenesis. Br J Cancer.

[B42] Teo NB, Shoker BS, Jarvis C, Martin L, Sloane JP, Holcombe C (2002). Vascular density and phenotype around ductal carcinoma in situ (DCIS) of the breast. Br J Cancer.

[B43] Siitonen SM, Kononen JT, Helin HJ, Rantala IS, Holli KA, Isola JJ (1996). Reduced E-cadherin expression is associated with invasiveness and unfavorable prognosis in breast cancer. Am J Clin Pathol.

[B44] Rakha EA, Abd El Rehim D, Pinder SE, Lewis SA, Ellis IO (2005). E-cadherin expression in invasive non-lobular carcinoma of the breast and its prognostic significance. Histopathology.

[B45] Asgeirsson KS, Jonasson JG, Tryggvadottir L, Olafsdottir K, Sigurgeirsdottir JR, Ingvarsson S, Ogmundsdottir HM (2000). Altered expression of E-cadherin in breast cancer. patterns, mechanisms and clinical significance. Eur J Cancer.

[B46] Yoshida R, Kimura N, Harada Y, Ohuchi N (2001). The loss of E-cadherin, alpha- and beta-catenin expression is associated with metastasis and poor prognosis in invasive breast cancer. Int J Oncol.

[B47] Camp RL, Rimm EB, Rimm DL (1999). Met expression is associated with poor outcome in patients with axillary lymph node negative breast carcinoma. Cancer.

[B48] Hayashida K, Johnston DR, Goldberger O, Park PW (2006). Syndecan-1 expression in epithelial cells is induced by transforming growth factor beta through a PKA-dependent pathway. J Biol Chem.

[B49] Yeaman C, Rapraeger AC (1993). Post-transcriptional regulation of syndecan-1 expression by cAMP in peritoneal macrophages. J Cell Biol.

[B50] Parker C, Rampaul RS, Pinder SE, Bell JA, Wencyk PM, Blamey RW, Nicholson RI, Robertson JF (2001). E-cadherin as a prognostic indicator in primary breast cancer. Br J Cancer.

[B51] Khoury H, Naujokas MA, Zuo D, Sangwan V, Frigault MM, Petkiewicz S, Dankort DL, Muller WJ, Park M (2005). HGF converts ErbB2/Neu epithelial morphogenesis to cell invasion. Mol Biol Cell.

[B52] Teo NB, Shoker BS, Jarvis C, Martin L, Sloane JP, Holcombe C (2003). Angiogenesis and invasive recurrence in ductal carcinoma in situ of the breast. Eur J Cancer.

[B53] Guidi AJ, Schnitt SJ, Fischer L, Tognazzi K, Harris JR, Dvorak HF, Brown LF (1997). Vascular permeability factor (vascular endothelial growth factor) expression and angiogenesis in patients with ductal carcinoma in situ of the breast. Cancer.

[B54] Heideman DA, Overmeer RM, van Beusechem VW, Lamers WH, Hakvoort TB, Snijders PJ, Craanen ME, Offerhaus GJ, Meijer CJ, Gerritsen WR (2005). Inhibition of angiogenesis and HGF-cMET-elicited malignant processes in human hepatocellular carcinoma cells using adenoviral vector-mediated NK4 gene therapy. Cancer Gene Ther.

[B55] Jeltsch M, Kaipainen A, Joukov V, Meng X, Lakso M, Rauvala H, Swartz M, Fukumura D, Jain RK, Alitalo K (1997). Hyperplasia of lymphatic vessels in VEGF-C transgenic mice. Science.

[B56] Kinoshita J, Kitamura K, Kabashima A, Saeki H, Tanaka S, Sugimachi K (2001). Clinical significance of vascular endothelial growth factor-C (VEGF-C) in breast cancer. Breast Cancer Res Treat.

[B57] Jakobsson L, Kreuger J, Holmborn K, Lundin L, Eriksson I, Kjellen L, Claesson-Welsh L (2006). Heparan sulfate in trans potentiates VEGFR-mediated angiogenesis. Dev Cell.

[B58] Wülfing P, Diallo R, Kersting C, Wülfing C, Poremba C, Greb RR, Böcker W, Kiesel L (2004). Endothelin-1, endothelin-A- and Endothelin-B-receptor expression in preinvasive and invasive breast disease. Oncol Rep.

[B59] Pennacchietti S, Michieli P, Galluzzo M, Mazzone M, Giordano S, Comoglio PM (2003). Hypoxia promotes invasive growth by transcriptional activation of the met protooncogene. Cancer Cell.

[B60] Haug C, Schmid-Kotsas A, Zorn U, Bachem MG, Schuett S, Gruenert A, Rozdzinski E (2000). Hepatocyte growth factor is upregulated by low-density lipoproteins and inhibits endothelin-1 release. Am J Physiol Heart Circ Physiol.

[B61] Rosano L, Spinella F, Di Castro V, Nicotra MR, Dedhar S, de Herreros AG, Natali PG, Bagnato A (2005). Endothelin-1 promotes epithelial-to-mesenchymal transition in human ovarian cancer cells. Cancer Res.

[B62] Rosano L, Spinella F, Di Castro V, Decandia S, Nicotra MR, Natali PG, Bagnato A (2006). Endothelin-1 is required during epithelial to mesenchymal transition in ovarian cancer progression. Exp Biol Med (Maywood).

[B63] Bagnato A, Rosano L, Spinella F, Di Castro V, Tecce R, Natali PG (2004). Endothelin B receptor blockade inhibits dynamics of cell interactions and communications in melanoma cell progression. Cancer Res.

[B64] Jamal S, Schneider RJ (2002). UV-induction of keratinocyte endothelin-1 downregulates E-cadherin in melanocytes and melanoma cells. J Clin Invest.

[B65] Bagnato A, Natali PG (2004). Endothelin receptors as novel targets in tumor therapy. J Transl Med.

[B66] Wakeling AE (2005). Inhibitors of growth factor signalling. Endocr Relat Cancer.

[B67] Ciardiello F, Troiani T, Caputo F, De Laurentiis M, Tortora G, Palmieri G, De Vita F, Diadema MR, Orditura M, Colantuoni G (2006). Phase II study of gefitinib in combination with docetaxel as first-line therapy in metastatic breast cancer. Br J Cancer.

[B68] Jimenez B, Volpert OV (2001). Mechanistic insights on the inhibition of tumor angiogenesis. J Mol Med.

[B69] Folkman J (2006). Angiogenesis. Annu Rev Med.

[B70] Tolgay Ocal I, Dolled-Filhart M, D'Aquila TG, Camp RL, Rimm DL (2003). Tissue microarray-based studies of patients with lymph node negative breast carcinoma show that met expression is associated with worse outcome but is not correlated with epidermal growth factor family receptors. Cancer.

[B71] Lengyel E, Prechtel D, Resau JH, Gauger K, Welk A, Lindemann K, Salanti G, Richter T, Knudsen B, Vande Woude GF (2005). C-met overexpression in node-positive breast cancer identifies patients with poor clinical outcome independent of Her2/neu. Int J Cancer.

[B72] Ghoussoub RA, Dillon DA, D'Aquila T, Rimm EB, Fearon ER, Rimm DL (1998). Expression of c-met is a strong independent prognostic factor in breast carcinoma. Cancer.

[B73] Nakopoulou L, Gakiopoulou H, Keramopoulos A, Giannopoulou I, Athanassiadou P, Mavrommatis J, Davaris PS (2000). c-met tyrosine kinase receptor expression is associated with abnormal beta-catenin expression and favourable prognostic factors in invasive breast carcinoma. Histopathology.

[B74] Heimann R, Lan F, McBride R, Hellman S (2000). Separating favorable from unfavorable prognostic markers in breast cancer: the role of E-cadherin. Cancer Res.

[B75] Charpin C, Garcia S, Bonnier P, Martini F, Andrac L, Choux R, Lavaut MN, Allasia C (1998). Reduced E-cadherin immunohistochemical expression in node-negative breast carcinomas correlates with 10-year survival. Am J Clin Pathol.

